# Orexins/Hypocretins: Gatekeepers of Social Interaction and Motivation

**DOI:** 10.3390/ijms25052609

**Published:** 2024-02-23

**Authors:** Sara Ouaidat, Inês M. Amaral, Diogo G. Monteiro, Hayat Harati, Alex Hofer, Rana El Rawas

**Affiliations:** 1 Division of Psychiatry I, Department of Psychiatry, Psychotherapy, Psychosomatics and Medical Psychology, Medical University Innsbruck, 6020 Innsbruck, Austria; 2 Neuroscience Research Center, Faculty of Medical Sciences, Lebanese University, Beirut P.O. Box 1533, Lebanon

**Keywords:** orexin, hypocretin, social interaction, reward, stress

## Abstract

Ever since the discovery of the brain’s orexin/hypocretin system, most research was directed toward unveiling its contribution to the normal functioning of individuals. The investigation of reward-seeking behaviors then gained a lot of attention once the distribution of orexinergic neurons was revealed. Here, we discuss findings on the involvement of orexins in social interaction, a natural reward type. While some studies have succeeded in defining the relationship between orexin and social interaction, the controversy regarding its nature (direct or inverse relation) raises questions about what aspects have been overlooked until now. Upon examining the literature, we identified a research gap concerning conditions influencing the impact of orexins on social behavior expression. In this review, we introduce a number of factors (e.g., stress, orexin’s source) that must be considered while studying the role of orexins in social interaction. Furthermore, we refer to published research to investigate the stage at which orexins affect social interaction and we highlight the nucleus accumbens (NAc) shell’s role in social interaction and other rewarding behaviors. Finally, the underlying orexin molecular pathway influencing social motivation in particular illnesses is proposed. We conclude that orexin’s impact on social interaction is multifactorial and depends on specific conditions available at a time.

## 1. Introduction

Social interaction (SI) is a critical event occurring among diverse species and involving the mutual communication between conspecifics to ensure their survival and continuity [1]. In fact, SI has two opposing faces, depending on the context in which it occurs [2]. When SI occurs in the context of drug use, it ultimately enhances the drug’s effects; however, if it is made available in a context distinct from that of drugs, it offers protection against substance abuse and drug addiction [2].

The rewarding properties of SI can be investigated using a behavioral paradigm called conditioned place preference (CPP) [3]. CPP is a kind of Pavlovian conditioning conducted in a CPP behavioral apparatus and involves establishing an association between a context and a stimulus by continuously exposing the animal to such stimulus while it is placed in a specific compartment. It consists of either two or three chambers that are distinguished by a number of visual cues as well as tactile cues [4]. During SI CPP, the natural preference of animals is determined by a pretest. Afterwards, animals are paired with sex and weight-matched conspecifics for half of the conditioning sessions, being alone in the opposite compartment for the remaining half of these sessions [5,6,7,8,9]. On the day of the test, animals are allowed to choose the preferred compartment of the CPP apparatus. If SI exerts its rewarding effects, the animal will most likely spend more time in the compartment associated with the SI stimulus and, consequently, exhibit a social phenotype.

Not every individual is able to experience SI rewards. For instance, Cremers et al. showed that individuals suffering from social anxiety lack the motivational preference for social rewards [10] and Chevallier et al. demonstrated the low social motivation experienced by autism spectrum disorder (ASD) patients [11]. Furthermore, SI may become severely impaired in cases of drug addiction and other substance use disorders (SUD) [12], such that SUD patients may tend to self-isolate and become completely withdrawn from the outside world.

The following unanswered questions were raised: (1) What factors contribute to individual differences across the distinct SI profiles and (2) Is it possible to induce a shift in such profiles via investigating the underlying neurological bases of different social phenotypes? Numerous research studies have attempted to find neurobiological determinants that might help to switch the non-social and passive coping behavior to a social and active coping behavior [13]. The present review focuses on the orexin/hypocretin system of the brain and how it contributes to SI.

Orexins, also known as hypocretins, are lateral hypothalamic neuropeptides that were discovered in 1998 by two independent groups [14,15]. In fact, due to the implication of this system in feeding behavior, the term orexin was derived from the Greek word ‘orexis’, which literally means ‘appetite’ [15]. In addition, the term ‘hypocretin’ was further established to confirm the localized expression of these neuropeptides in the hypothalamus and due to their structural homology with the ‘secretin’ molecule [14]. Furthermore, orexins or hypocretins have been classified into two categories: orexin A, also called hypocretin 1 (OXA/HCRT1), and orexin B or hypocretin 2 (OXB/HCRT2), both of which are derived from a common precursor called prepro-orexin, which is selectively expressed in the lateral hypothalamus [16]. Concerning their binding affinities, OXA has been shown to bind with higher affinity to orexin 1 receptors (OX1R), whereas OXB has a greater binding affinity to orexin 2 receptors (OX2R) [15]. Interestingly, both orexin types, with their corresponding receptors, have been linked with several physiological functions, including the regulation of sleep and wakefulness [17]. Indeed, several studies have demonstrated that the administration of orexins, in particular OXA, promotes wakefulness and suppresses rapid eye movement (REM) sleep [18,19], leading to sleep disturbances [20,21]. Moreover, impaired sleep has been associated with the poor social behavior observed among children and adolescents [22]. Thus, when investigating orexin’s impact on SI, it is crucial to address changes in sleeping patterns. Orexins also contribute to cardiovascular regulation [23]. Indeed, orexin knockout mice showed diminished cardiovascular responses in a resident–intruder model of SI stress [24].

In adult rats, both orexin receptor mRNAs are found in higher concentrations in the brain than in any other organ [15]. Nevertheless, the distribution of these receptors varies across different brain regions. For instance, OX1Rs are highly expressed in the bed nucleus of the stria terminalis (BNST), the amygdala, the locus coeruleus (LC), the laterodorsal tegmental nucleus, and the pedunculopontine tegmental nucleus (LDT/PPT). OX2Rs are selectively expressed in the nucleus accumbens (NAc) and within the tuberomammillary nucleus (TMN). Furthermore, among others, both receptor types are co-localized in brain regions like the medial prefrontal cortex (mPFC), the hippocampus, the hypothalamic paraventricular nucleus (PVN), the paraventricular nucleus of the thalamus (PVT), and the ventral tegmental area (VTA) of the midbrain [25].

Given the distribution of orexin neurons in reward-related areas of the brain, one can infer their function and implication in reward as well as motivation [26,27]. Numerous studies using animal models have confirmed the implication of the orexin system in the modulation of hedonic behaviors such as those associated with the intake of food or drugs of abuse [28]. In fact, orexin might enhance such hedonic behaviors via boosting the sensation of pleasure or reward in response to diverse stimuli [29]. Thus, the orexin system is an important candidate for the investigation and further analysis of many rewarding behaviors, including SI. In addition to their role in reward and motivation, orexins have been classified as novel therapeutic targets in inflammatory and neurodegenerative diseases due to their anti-inflammatory and neuroprotective properties [30]. This issue, indeed, is highly compelling, especially since inflammation has been associated with psychosis, stress, and social deficits [31].

A number of previous research studies aimed to assess the relationship between orexin and social reward. For instance, Faesel et al. explored how orexin deficiency affects the social behavior of mice [32]. Surprisingly, only female orexin-deficient mice showed decreased sociability, as well as reduced preference for social novelty, compared to the wild-type littermates [32]. Furthermore, OX1R-deficient mice showed reduced sociability [33] and mice, whose orexin neurons degenerate at the age of three months, displayed impairments in social memory, which could be ameliorated by nasal application of OXA [34]. Moreover, optogenetic inhibition of OX1R disrupted social behavior in male mice [35]. In contrast, others have demonstrated that OXA levels are highest during the perception of positive emotions, SI, and anger [36]. Overall, these studies suggest a direct correlation (high orexin = increased SI or contrariwise) between the two variables, which we believe to be the case in the absence of stress. Recently, we have demonstrated the presence of comparable orexin levels in both male and female mice with distinct SI profiles [37]. Our finding was in accordance with Reppucci’s study showing that rats, whether exposed or not to a 10 min social play test, had exactly the same total amount of OXA neurons, but differed in terms of neuron activation [38].

In the following review, we suggest the implication of orexins in SI when related to stress. We will also discuss how distinct sources of orexins (endogenous vs. exogenous) affect SI. Additionally, we will refer to published research to further investigate the stage at which orexin contributes to social behavior expression and we will highlight the role played by the NAc shell in SI and other rewarding behaviors. Last but not least, we will shed light on possible molecular pathways through which orexins may impact social motivation.

## 2. Implication of Orexin in Social Behaviors upon Stress Exposure

High arousal states, particularly stress, are modulated by the well-known lateral hypothalamic neuropeptides, orexins. Indeed, Berridge and España have previously demonstrated the importance of orexins in response to stressful stimuli [39], with significant differences depending on the stressor type [40]. Anatomically, orexins and their corresponding receptors are found in both central structures involved in the regulation of the hypothalamo–pituitary–adrenal (HPA) axis (hippocampus, amygdala, and the paraventricular nucleus of the hypothalamus) and peripheral HPA axis sites (pituitary and adrenal glands) [41]. Functionally, an intracerebroventricular infusion of orexins leads to an activation of the HPA axis, resulting in increased corticotropin-releasing hormone (CRH) production in the hypothalamus, and, consequently, a release of adrenocorticotropin (ACTH) and corticosterone from the pituitary and adrenal glands, respectively [41,42,43,44].

Data throughout the literature link high orexin levels with increased susceptibility to drug addiction [45], higher levels of stress [46], and decreased SI in the presence of stress [47]. Grafe and colleagues hypothesized that lower orexin levels may contribute to resilience against repeated social stress [48]. To test this hypothesis, Sprague Dawley rats were segregated into two distinct phenotypes, the active and the passive coping ones, using a 5-day social defeat paradigm, which was based on a resident–intruder model. Afterwards, designer receptor exclusively activated by designer drugs (DREADDs) were utilized for further inhibition of orexin neurons prior to each social defeat for 3 additional days. Remarkably, the prepro-orexin mRNA level was significantly lower in actively coping rats compared to the passively coping (vulnerable) ones. Thus, once orexin neurons were inhibited via DREADDS, an increase in SI and a decrease in depressive-like behaviors (decrease in immobility time during forced swim test) were observed in the vulnerable population of rats [48]. These findings suggest that resilience to social defeat can be promoted via a reduction in orexin levels and the orexin system may therefore serve as a significant target for the treatment of many stress-related disorders [48]. In addition, Eacret and colleagues demonstrated the induction of a passive coping behavior (decrease in the average latency to become defeated across five days) upon the activation of orexin neurons prior to each social defeat using DREADDS [47]. Interestingly, orexin stimulation during social defeat stress induced a reduction in SI in adult male rats [47]. Lastly, optogenetic stimulation of orexin neurons attenuated the time spent in SI, indicative of an animal’s increased anxiety state, while increasing the locomotor activity and the frequency of entries into the SI zone [49].

Based on the following data, it is possible to suggest the implication of orexins in SI when stress becomes involved. Interestingly, an inverse relationship between orexins and SI in the presence of stress may only exist in male, but not in female animals (refer to Table 1 for a summary of all studies investigating the orexin-SI relationship). Thus, we believe that the nature of this relationship not only depends on the presence/absence of a stress factor, but also on the sex of the investigated species. Indeed, according to a study done by Jöhren and colleagues, the orexinergic system was shown to be differentially influenced by sex steroids [50]. In female rats that underwent ovariectomy, estradiol treatment was found to abolish the elevated levels of pituitary OX1R and adrenal OX2R mRNAs. Nonetheless, in orchidectomized males, the effect of testosterone treatment differed by reducing the increased OX1R mRNA while increasing the attenuated OX2R mRNA levels caused by the orchidectomy [50].

## 3. Exogenous vs. Endogenous Orexins: Impact on SI

Distinct sources of orexins are thought to differentially affect SI. These sources could be endogenous (within the animal) or exogenous (external source). Reppuci’s team, for instance, investigated the role of the orexin/hypocretin system in the expression of juvenile social play behavior in rats [38]. In this study, juvenile rats were segregated into two groups, the social play group and the no social play control group. After 10 min of social play testing, Fos-induction of OXA neurons (marker of neuronal activity) was measured across the two groups. In addition, they investigated the impact of OXA on social play expression. One of their main findings was that the central administration of OXA led to a reduction in the time spent in social play as well as allogrooming behaviors in both males and females [38]. In addition, other researchers used the social affective preference (SAP) test to explore the possible neural mechanisms involved in shaping social behavior [53]. It was shown that the microinjection of OXA in the insular cortex had the capacity to increase the interaction time in the one-on-one SI test with both juvenile and adult naïve conspecifics [53].

Ji and colleagues assessed the effect of orexins on SI between novel partners using a behavioral paradigm called the social proximity test [54]. Social proximity was evaluated depending on the time the head of the animal stayed near a cage beholding a novel partner and the number of times that the forepaws climbed the cage. Interestingly, the time spent in exploring the area close to a caged novel rat, as well as the number of times in climbing this cage, significantly increased upon a bilateral microinjection of OXA in the ventral pallidum (VP) of rats. However, this was not the case reported for endogenous orexins, where OX1R knockdown via shRNA lentivirus had no impact on novel SI. Given such contradictory effects of distinct orexin sources on novel SI and their consistent impact on forced swimming and sucrose preference, Ji et al. additionally investigated how endogenous orexins might act in response to stress. It was shown that endogenous orexins within the VP are critical for alleviating social avoidance only in the case of stressful conditions [54]. This, indeed, confirms our point of view discussed earlier in Section 2. Nevertheless, decreased sociability and reduced preference for social novelty were found only in female, but not male orexin-deficient mice [32]. Therefore, it seems that endogenous orexin’s effect on SI depends on the sex of the investigated species, being more prevalent in females.

In conclusion, the role of orexins in the establishment of social behaviors depends particularly on its origin. It appears that external sources of orexins (obtained via microinjections, central administration, etc.) have the capacity to alter social behavior expression while the impact of endogenous sources varies depending on the conditions (e.g., sex, presence/absence of stress, the methodologies used for social behavior assessment, etc.) (refer to Table 2 for a summary). Thus, further research investigating different stress conditions using male and female animals, as well as assessing different aspects of social behaviors, is required to elucidate the role of endogenous orexin on SI.

## 4. Involvement of Orexins in SI Depends on the Memory Phase

The orexin/hypocretin system has been shown to be indispensable for a particular aspect of SI referred to as the ‘social memory’ [34]. Indeed, Yang and colleagues assessed the effect of orexin on sociability, social novelty, as well as on social recognition memory using adult orexin/ataxin-3-transgenic (AT) mice, in which Hcrt neurons degenerate by 3 months of age. Surprisingly, AT mice exhibited normal sociability and preference for social novelty compared to their wild-type littermates. Although AT mice developed deficits in long-term social memory, nasal administration of exogenous HCRT 1 had the potential to restore social memory in these mice [34]. Thus, the hypocretin system is not necessarily involved in the initiation of social approach, but has an important role in social memory [34]. This outcome might be attributed to the fact that orexin neurons constitute cell assemblies, which are cells linked to each other by reciprocal connections based on Hebb’s postulate of “neurons that fire together, wire together” [55], and which consequently participate, along with other neurotransmitters, in the formation of memory engrams via the consolidation of memory traces [56]. We hypothesize that when hypocretin neurons become destroyed in AT mice, a distortion may happen in the corresponding engram and that deficits in long-term social memory may be observed. It is plausible to suggest that hypocretin administration aids in memory restoration via activating other neuronal cells that are part of the same engram. More research is needed to confirm the suggested hypothesis.

A large number of studies point to the relevance of stress in the establishment of long-lasting memories. According to Roozendaal et al., stress hormones (mainly glucocorticoids) exert their memory-enhancing properties during a particular memory phase called memory consolidation [57]. As a matter of fact, the effects of glucocorticoids on memory consolidation are dependent on the noradrenergic activation of the BLA, as well as on interactions with other brain regions [57,58]. In addition, Wichmann and colleagues revealed the involvement of the NAc shell’s noradrenergic system in the enhancement of memory consolidation upon interacting with glucocorticoids [59]. Moreover, several studies have proven the existence of an interaction between the orexinergic and the adrenergic systems [26,60].

**Figure 1:**
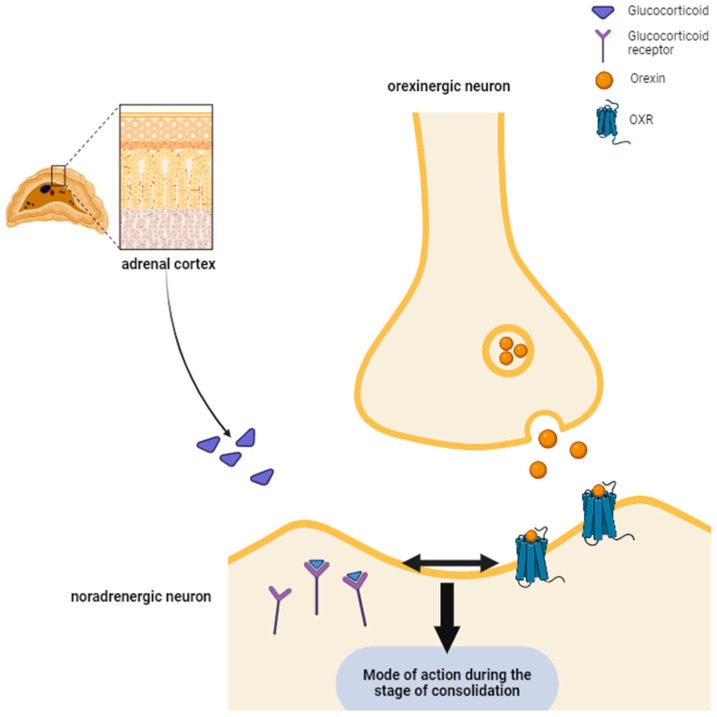
The indirect relation between glucocorticoids (GR) and the orexinergic system implies both of their effects exerted during memory consolidation. Both GRs and orexins mediate their mode of action during the stage of memory consolidation. GRs released from the adrenal cortex interact with their corresponding receptors expressed on noradrenergic postsynaptic membranes in order to exert their memory-enhancing properties at the stage of memory consolidation. Due to the existence of orexinergic–noradrenergic interactions, the impact of orexins on social memory is also thought to occur during consolidation, similar to the effects of GRs.

Based on the presented data, it seems that there might exist an indirect relation between stress hormones (e.g., glucocorticoids) and the orexin system. In that sense, as stress hormones exert their action (memory-facilitating) during the phase of memory consolidation, we believe that the effect of orexins on social memory occurs once it has reached the stage of consolidation (Figure 1).

## 5. The Nucleus Accumbens Shell: A Pivotal Role in SI and Reward-Seeking Behavior via Orexins

The NAc is a part of the mesolimbic system receiving dopaminergic projections from the VTA [61]. In fact, the NAc consists of two anatomically and chemically distinct areas: the NAc core and the NAc shell [62,63]. Data throughout the literature have confirmed the existence of shell–core differences in terms of the distribution of several neurobiological molecules and their corresponding fibers, including the orexin immunoreactive fibers, with a relative higher density in the shell compared to the core [64].

The importance of the NAc shell in the processing of natural rewards, like SI, has been previously highlighted [65]. In one study, Amaral et al. demonstrated that SI reward in rats induced an increase in the calcium/calmodulin-dependent protein kinase II (CaMKII) in the NAc of the brain. Thus, when CaMKII in the NAc shell was blocked by KN-93 (CaMKII inhibitor), a shift in preference toward cocaine was observed in a concurrent CPP where SI was available as an alternative to this drug [66]. In addition, Salti and colleagues investigated the activity of p38-mitogen-activated protein kinase (MAPK), also known as the stress-activated protein kinase (SAPK), in the NAc of rats expressing either cocaine or SI CPP and in saline controls. Surprisingly, rats expressing SI CPP experienced a reduction in pp38 (active/phosphorylated form of p38 MAPK) neuronal levels in the NAc shell 24 h following SI to the level of naïve untreated rats [67]. This is in line with our previous finding [68], where we showed that SI reward possesses anti-stress effects. Hence, it appears that SI reward in the NAc shell has a dual action: an increase in reward via CaMKII and a decrease in stress via P38 MAPK [66]. Moreover, the differential effects of lesions in the NAc core and shell on CPP for cocaine versus SI were explored by Fritz and colleagues. The inactivation of the NAc shell shifted preference toward cocaine, unlike the lesioning of the core that resulted in a shift in CPP toward SI [69]. In addition, activation of the NAc shell in rats was associated with operant social seeking to a novel partner following social isolation [70].

It should be noted that the NAc shell participates in neural mechanisms in which orexin regulates reward-seeking behaviors [71]. For instance, the role of OX1Rs in driving addictive behaviors, especially excessive alcohol drinking, was explored in C57BL/6 mice [72]. Interestingly, the inhibition of OX1Rs in the medial NAc shell significantly decreased alcohol intake, thereby confirming OX1R’s role in promoting excessive alcohol drinking [72]. In addition, local OXA has been shown to be a critical modulator of dopamine within the NAc shell region and is therefore essential for reward-seeking behavior [73]. In another study, a morphine CPP model was utilized to investigate the potential involvement of orexin receptors within the NAc shell in stress- and drug-priming-induced CPP reinstatement [74]. Inhibition of OX1Rs and OX2Rs in the NAc shell significantly decreased stress—but not drug-priming-induced morphine CPP reinstatement. Therefore, orexin receptors in the NAc shell have a central role in the stress-induced relapse of opioid seeking [74].

Overall, these data confirm the relevance of the NAc shell in SI as well as other rewarding behaviors, including drug-seeking behaviors. More research is needed to investigate the potential mechanisms by which the orexinergic contents of the NAc shell contribute to SI reward, as most of the studies focused on the implication of the NAc shell’s orexinergic system on drug rewards rather than on natural reward types.

## 6. How Orexin Impacts Social Motivation: Insight on a Potential Molecular Pathway

As previously mentioned, many patients suffering from SUD, neuropsychiatric illnesses, or neurodevelopmental diseases tend to experience reduced social motivation. From here, we might ask ourselves the following questions: (1) How does the orexin system affect social motivation in these patients? (2) At which stage of the molecular pathway is this effect exerted?

**Figure 2:**
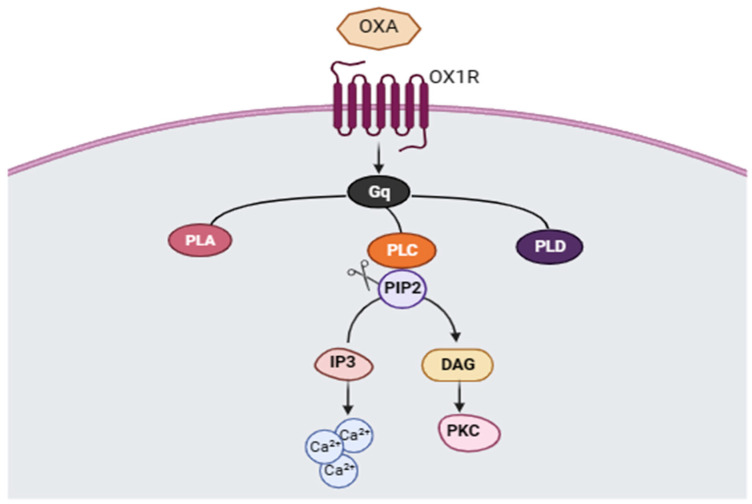
Signal transduction cascade initiated by orexin A (OXA)/orexin 1 receptor (OX1R) coupling. The binding of OXA to OX1R triggers Gq protein activation which, in turn, induces the activation of phospholipases A (PLA), D (PLD) and C (PLC). The PLC pathway involves the cleavage of phosphoinositol-diphosphate (PIP2) into inositol-triphosphate (IP3) and diaminoglycerol (DAG). IP3 and DAG influence the intracellular calcium (Ca^2+^) concentration by triggering the release of Ca^2+^ from intracellular stores and stimulating protein kinase C (PKC) activity, respectively.

To answer these questions, we must first understand the underlying signal transduction cascade mediated by orexin and its receptors. Depending on the G protein-coupled receptor (GPCR) being activated (OX1R or OX2R), distinct molecular pathways are transduced. For instance, the coupling between OXB and OX2R induces the elevation of postsynaptic intracellular calcium concentration ([Ca^2+^]i) via adenylyl cyclase-protein kinase A mediated activation of the R- and T-type voltage-gated calcium channels [75]. As for the OXA neuropeptide, its binding to OX1R influences intracellular Ca^2+^ signaling via the phospholipase C (PLC) cascade (PLC-IP3/DAG) [76], activating a G protein subtype called ‘Gq’, which, in turn, stimulates the subsequent activation of several phospholipases, including phospholipases A (PLA), D (PLD)s and C (PLC). As a matter of fact, PLC acts by cleaving phosphoinositol-diphosphate (PIP2) into inositol-triphosphate (IP3) and diaminoglycerol (DAG) second messengers, which subsequently trigger the release of Ca^2+^ cations from intracellular stores and stimulate protein kinase C (PKC) activity, respectively [77] (Figure 2). In the present section, we are going to focus on PKC activity, a downstream target of orexin pathway activation, and the reason behind highlighting it as a potential molecular underpinning of social motivation.

ASD patients are known to experience diminished social motivation [11]. According to Messina and colleagues, orexin A levels tend to be higher among patients with ASD compared to healthy individuals [78]. In addition, a number of studies highlighted the existence of dopamine circuitry dysfunctions in neurodevelopmental illnesses like ASD [79]. Not only did autistic children experience diminished dopaminergic activity within the mPFC, but they also had deficits in the NAc neural response to social rewards [80,81]. According to published data, orexins stimulate dopaminergic cell-firing and enhance the release of dopamine under two main circumstances: either in case of normal baseline conditions or in response to drugs of abuse [82,83]. Thereby, the direction of such a relationship might vary outside those specific conditions. Here, for example, we suggest that when OXA is high, dopamine becomes low, and this might contribute to the low social motivation exhibited by these patients. Why is this happening? As mentioned earlier, when OXA interacts with its corresponding receptors, the PLC signal transduction cascade potentially becomes stimulated and, thereby, an activation of PKC is observed. From this perspective, it seems that the elevation of OXA levels seen in ASD indirectly induces the over-activation of PKC within postsynaptic cells, including those expressing dopaminergic receptors. Indeed, our point of view is reinforced by one particular study reporting on the emergence of behavioral abnormalities (e.g., impaired habituation learning) commonly seen in ASD, caused by PKC hyper-activation in zebrafish, thus implying the involvement of the PKC pathway in the pathogenesis of ASD [84].

How might PKC’s activity affect social motivation in ASD? PKC is a kinase that has been demonstrated to play a role in dopamine D2 receptor desensitization and internalization via its phosphorylating activity [85]. Concerning the function of dopamine receptors, dopamine D2 receptors in the NAc were found to drive social attachment in female prairie voles [86]. In addition, enhanced motivation for rewards was reported upon an increase in D2 receptor expression within the adult NAc region of C57Bl/6J mice [87]. In a study conducted by Zhang’s team, D2 receptors within the NAc shell were found to be reduced following chronic social isolation, and the behavioral abnormalities caused by this isolation were reversed upon the injection of D2 receptor agonist into the NAc shell [88]. Furthermore, many studies revealed the importance of dopamine in the motivation to seek rewards [89,90]. Therefore, dopaminergic receptor-signaling might be critical for social motivation.

**Figure 3:**
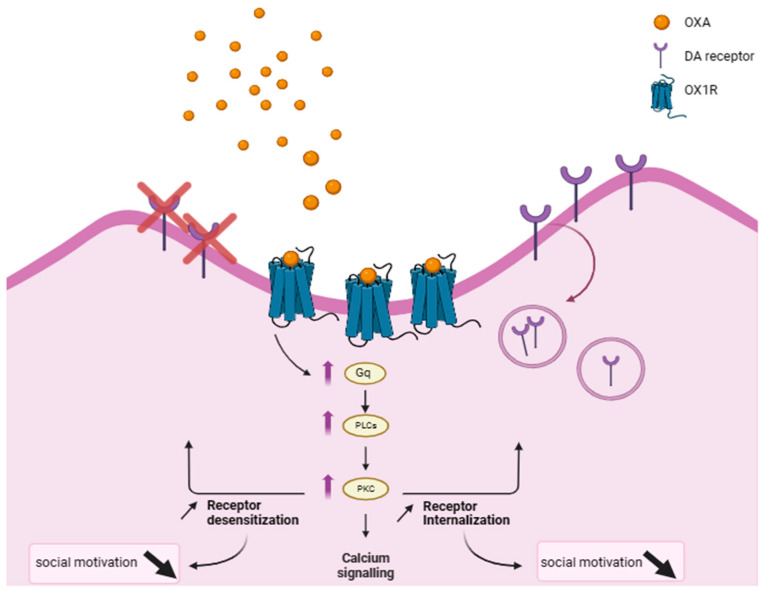
Orexin’s effect on social motivation in autism spectrum disorder (ASD). High orexin A (OXA) levels are reported in ASD. The increased coupling between OXA and its corresponding receptors (OX1R) contributes to Gq (globular protein q) hyperactivation and subsequently to an increase in phospholipase C (PLC) activity. This increased PLC activity triggers an over-activation in a particular kinase called protein kinase C (PKC). Eventually, the hyperactivity of PKC stimulates massive dopamine (DA) receptor internalization and/or desensitization, which might explain the diminished social motivation observed in ASD patients.

According to the presented data, the hyperactivity of PKC in ASD is thought to contribute to the internalization of a substantial amount of dopamine D2 receptors and/or massive receptor desensitization on postsynaptic membranes. This, in our opinion, might explain the low social motivation observed in these patients (Figure 3). More research is needed to further investigate the possible involvement of different orexin-signaling pathways in social motivation/interaction across many other related illnesses.

## 7. Conclusions

Switching non-social and passive coping behavior into social and active coping behavior remains the biggest challenge that neuropsychiatrists face when dealing with patients suffering from psychiatric disorders, including drug-related diseases, where SI becomes impaired. Over decades, many studies have attempted to find neurobiological correlates that might help to accomplish this switch. In the present review, we focused our attention on a particular candidate, the orexin/hypocretin system of the brain. We believe that, by better understanding (1) the differences between the distinct sources of orexins (endogenous vs. exogenous), (2) how orexin contributes to SI, (3) the stage at which orexin influences social behavior expression, (4) the implication of brain regions (e.g., the NAc shell) in SI, and (5) the underlying orexin molecular pathways, we will be able to unravel the cause of SI deficits in many illnesses and consequently help to reverse and/or minimize this impairment via targeting the underlying molecular candidates.

## Figures and Tables

**Table 1:** Summary of the findings investigating the relation between orexins and social interaction (SI). OXA, orexin A; OX1R−/−, orexin 1 receptor deficit; ACC, anterior cingulate cortex; Stress: + presence, − absence.

Study	Molecular	Relation	Stress	Results in	Reference
Orexin signaling during social defeat stress influences subsequent SI behavior and recognition memory	Orexin stimulation, decreased SI	Inverse	+	Males	[47]
Reduced orexin system function contributes to resilience to repeated social stress	Orexin inhibition, increased SI Low prepro-orexin mRNA in resilient rats	Inverse	+	Males	[48]
Optogenetic examination identifies a context-specific role for orexins/hypocretins in anxiety-related behavior	Orexin photostimulation, reduced SI time	Inverse	+	Males	[49]
Involvement of orexin/hypocretin in the expression of social play behavior in juvenile rats	OXA administration, decreased social play	Inverse	−	Males, females	[38]
Orexin deficiency affects sociability and the acquisition, expression, and extinction of conditioned social fear	Orexin deficiency, reduced sociability and preference for social novelty in female mice	Direct	−	Females	[32]
Comprehensive behavioral analysis of male Ox1r (−/−) mice showed implication of orexin receptor-1 in mood, anxiety, and social behavior	OX1R−/− mice, decreased SI, increased anxiety-like behavior	Direct	−	Males	[33]
Hypocretin/orexin neurons encode social discrimination and exhibit a sex-dependent necessity for social interaction	OX1R blockade, reduced SI in male, but not female mice	Direct	−	Males	[35]
Molecular and cellular mechanisms for differential effects of chronic social isolation stress in males and females	OXA treatment in female stressed mice, social withdrawal rescued	Direct	+	Females	[51]
Anterior cingulate cortex orexin-signaling mediates early-life stress-induced social impairment in females	ACC orexin terminal activation, diminished sociability rescued	Direct	+	Females	[52]
Hypocretin/orexin neurons contribute to hippocampus-dependent social memory and synaptic plasticity in mice	OXA nasal administration, social memory restored	Direct	−	Males, females	[34]
Human hypocretin and melanin concentrating hormone levels are linked to emotion and SI	OXA levels maximal during SI, positive emotions and anger	Direct	−	−	[36]

**Table 2:** Table summarizing the distinct modes of action exerted by exogenous and endogenous sources of orexins on social interaction (SI) expression. OXA: orexin A, OX1R: orexin 1 receptor.

Orexin’s Source	Mode of Action	References	Conclusion
Exogenous	OXA administration, reduced social play time and allogrooming behaviors	[38]	
	OXA microinjection, increased interaction time in one-on-one SI test	[53]	Exogenous sources are capable of the alteration of social behavior expression
	OXA bilateral microinjection, increase in the time spent in exploring the area close to a caged novel rat and the number of times climbing this cage	[54]	
Endogenous	OX1R knockdown, no effect on novel SI In case of stress, alleviation of social avoidation by endogenous orexin	[54]	The impact of endogenous orexins on SI varies depending on certain conditions, namely: stress, sex of the investigated species, methods used for social behavior assessment, etc.
	Decreased sociability and preference for social novelty observed only in female, but not male orexin-deficient mice	[32]	

## Data Availability

No new data were created or analyzed in this study. Data sharing is not applicable to this article.
